# Acupoint injection combined with pelvic floor rehabilitation in the treatment of postpartum pelvic floor disorders: A protocol of randomized controlled trial

**DOI:** 10.1097/MD.0000000000025511

**Published:** 2021-06-11

**Authors:** Ying Zheng, Hongying Yang, Xunfu Yin, Xiujuan Ma, Llihua Guo

**Affiliations:** a Chongqing Ninth People's Hospital; b College of Artificial Intelligence, Southwest University, Chongqing, China.

**Keywords:** acupoint injection, combined therapy, pelvic floor dysfunction, pelvic floor rehabilitation, protocol, randomized controlled trial

## Abstract

**Background:**

Female pelvic floor dysfunction is one of the common chronic diseases affecting women's physical and mental health. Pregnancy and delivery are one of the main causes. Pelvic floor rehabilitation is a common method for the treatment of postpartum pelvic floor dysfunction, but it has some defects. Acupoint injection has advantages in the treatment of postpartum pelvic floor dysfunction, but there is a lack of standard clinical research to verify it. Therefore, the purpose of this randomized controlled trial is to evaluate the efficacy and safety of acupoint injection combined with pelvic floor rehabilitation in the treatment of postpartum pelvic floor disorders.

**Methods:**

This is a prospective randomized controlled trial to study the efficacy and safety of acupoints injection combined with pelvic floor rehabilitation. And it is approved by the Ethics Committee of Clinical Research of our hospital. Patients were randomly divided into observation group (acupoint injection combined with pelvic floor rehabilitation group) or control group (pelvic floor rehabilitation group alone). The patients were followed up for 8 weeks after 12 weeks of treatment. The observation indexes included: pelvic organ prolapse degree, pelvic floor muscle strength, urinary incontinence score, adverse reactions, among others. Data were analyzed using the statistical software package SPSS version 18.0.

**Conclusions:**

This study will evaluate the efficacy and safety of acupoint injection combined with pelvic floor rehabilitation in the treatment of postpartum pelvic floor dysfunction, and provide reliable reference for the clinical application of this project.

**Trial registration:**

OSF Registration number: DOI 10.17605/OSF.IO/VC65Z

## 1 Introdoction

Pelvic floor dysfunction (PFD) refers to a group of diseases caused by weak supporting tissue of the pelvic floor, including pelvic organ prolapse (POP), stress urinary incontinence, sexual dysfunction, and so on.^[1]^ In China, the incidence of PFD in women is as high as 58.7%, of which POP is the most common (48.73%), followed by stress urinary incontinence (8.7%).^[2]^ Although PFD is not like a malignant tumor endangering the lives of women, because of the special nature of PFD, patients often suffer from great psychological pressure,^[3]^ It has become 1 of the 5 most common chronic diseases affecting women's physical and mental health and quality of life.^[4]^

The pathogenesis of PFD is complex. Previous clinical studies have shown that pelvic floor muscle injury and pelvic floor connective tissue relaxation caused by various causes are its main pathogenesis.^[5]^ Pregnancy and delivery can lead to impaired pelvic floor muscle function, reduced pelvic floor muscle strength, and further cause urinary incontinence, pelvic organ prolapse, and other pelvic floor dysfunction diseases.^[6]^

Acupoint injection is a kind of alternative therapy based on Chinese meridian theory, which injects drugs into specific acupoints and achieves the purpose of treatment by stimulating acupoints.^[7]^ It is found that acupoint injection can improve the function of pelvic floor circulation, improve muscle strength and muscle tension, and accelerate nerve conduction.^[8]^ Pelvic floor rehabilitation training is a first-line conservative treatment for the prevention and treatment of pelvic floor disorders.^[9]^ By exercising the muscles, the patient can increase the muscle volume and raise the levator plate and genital hiatus, thus improving the support of pelvic organs.^[10]^ At present, there is a lack of clinical research on acupoint injection combined with pelvic floor rehabilitation in the treatment of postpartum pelvic floor disorders. Therefore, we designed this randomized controlled trial to evaluate the efficacy and safety of acupoint injection combined with pelvic floor rehabilitation in the treatment of postpartum female pelvic floor disorders.

## 2 Materials and methods

### 2.1 Study design

**Figure 1:**
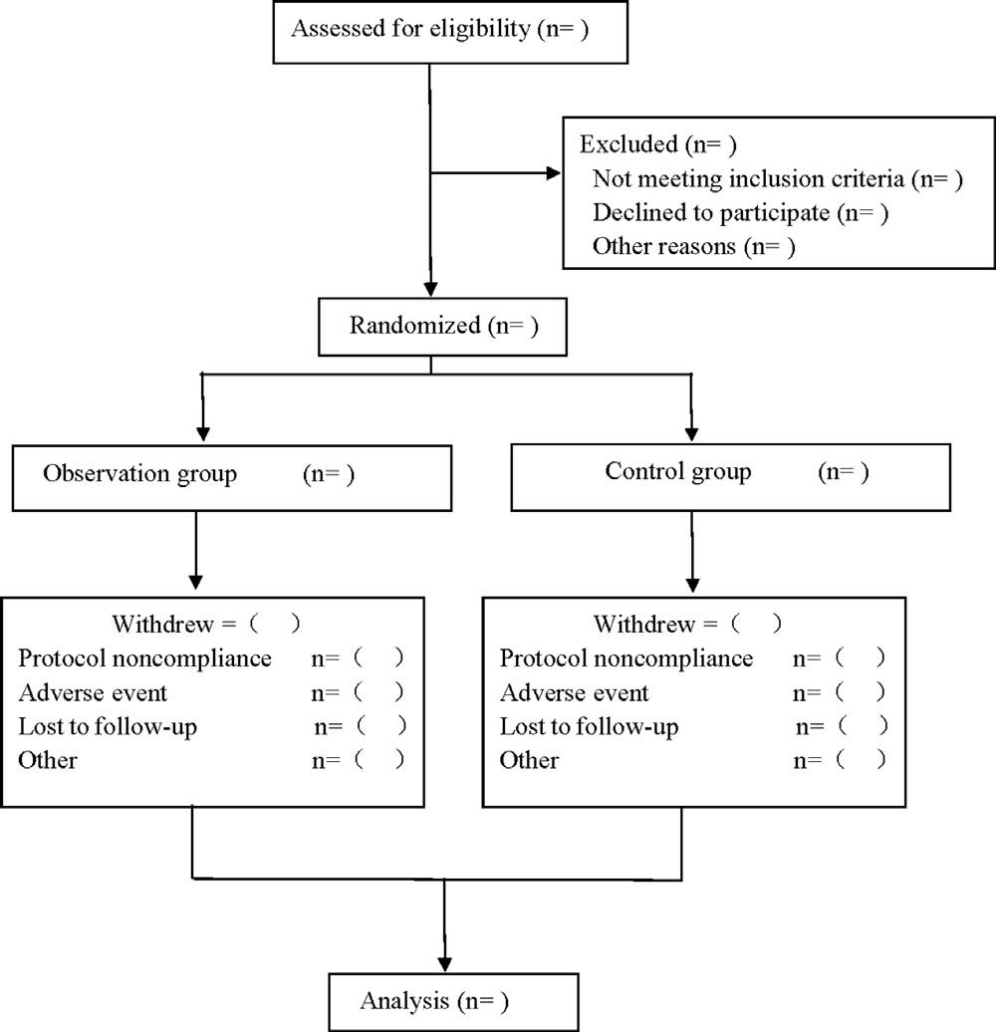
Flow diagram.

This is a prospective, single-center, randomized controlled trial to study the efficacy of pelvic floor rehabilitation combined with acupoint injection in the treatment of postpartum pelvic floor disorders. It was approved by the Ethics Committee of Traditional Chinese Medicine. This trial will follow the comprehensive trial reporting standard,^[11]^ and comply with Standard Protocol Recommendations for Interventional Trials 2013 Statement.^[12]^ The flow chart of the study is shown in Figure 1.

This research scheme is in line with the Helsinki Declaration and approved by the Clinical Research Ethics Committee of our hospital. This laboratory is registered in the open science framework (OSF) (registration number: DOI 10.17605/OSF.IO/VC65Z). Before being randomly divided into groups, all patients need to sign a written informed consent form, and they are free to choose whether or not to continue the trial at any time.

### 2.2 Patients

Inclusion criteria were: age 22 to 45 years’ old; Primipara who is full-term and vaginal delivery; the examination was in accordance with the diagnostic criteria of PFD within 180 days after delivery; pelvic floor muscle ≤III level, POP international quantitative framework dividing ≤II degrees; participate voluntarily and sign the informed consent form.

Exclusion criteria were: patients with previous history of reproductive system surgery; patients with neurological diseases, respiratory dysfunction, and vital organ dysfunction; patients with PFD disease before pregnancy; patients with a history of severe uterine malformation; patients with allergy or contraindications to the medication used in this study.

### 2.3 Sample size

The calculation of sample size is based on mean and standard deviation of muscle strength scores of class I muscle fibers in pelvic floor muscles after treatment. According to the results of the pilot study, the experimental group was 19.37 ± 2.26, and the control group was 21.01 ± 2.57. Set α = 0.025, unilateral test, β = 0.20. According to the calculation of PASS15.0 software, 36 participants are needed in each group; the estimated withdrawal rate is 10%, so that 40 participants will be included in each group.

### 2.4 Randomization and blinding

In this study, patients meeting the research criteria were included through pre-hospital recruitment and inpatient screening. The random number generator in Microsoft Excel was used to randomly divide the patients into the observation group and the control group according to the proportion of 1:1. The clinical research coordinator entered participant information on the tablet and was given a random number. The research assistant got the allocation of participants from the computer. Throughout the study, the research assistant was responsible for screening, recruiting participants, and assigning random numbers to participants who had been included. Outcome evaluator was responsible for the evaluation of the scale. Given the practical nature of acupoint injection, participants and practitioners might be aware of random assignment. However, the evaluators of the research results, the statisticians of data statistics and analysis did not know about the distribution.

### 2.5 Study design

#### 2.5.1 Pelvic floor rehabilitation

Pelvic floor rehabilitation consisted of 10 maximal voluntary contractions maintained for at least 6 seconds. At the end of a set of 10 contractions, 5 rapid contractions were performed. The interval between contractions was 6 seconds. This training can be done in lying, seated, kneeling on all fours, and standing positions. Two professional physiotherapists will provide face-to-face coaching training to the participants twice a week for 12 weeks.

In addition to completing face-to-face training with the therapist twice a week, all participants will be asked to follow the written instructions at home every day and record the frequency. A monitoring meeting will be held once a week, and professional physiotherapists will conduct new assessments and guidance according to the intensity, frequency, and timing of the participants’ training.

#### 2.5.2 Acupoint injection

The patient took a suitable position, exposed the skin of abdomen, waist and leg and located Guanyuan, Zusanli, and Sanyinjiao. After local disinfection, a 10 mL syringe was used to connect the No. 7 needle, and the needle was punctured slowly at the vertical acupoints. After the patient felt the sensation of acid swelling and no blood return after aspiration. Inject the medicine and about 0.5 to 1 mL was injected into each point. The acupuncture depth was about 2 to 3 cm. Drug composition: Huang Qi injection (China Wuxi SFDA approval number: Z32021030). After the injection, lie prone and rest for 30 minutes to observe whether the patient felt uncomfortable. The injection procedure was performed by the same doctor with >5 years of acupoint injection experience.

The observation group was combined with acupoint injection on the basis of pelvic floor rehabilitation training, whereas the control group was simply given rehabilitation training.

### 2.6 Evaluation criteria and judgment of curative effect

#### 2.6.1 Measurement of the degree of pelvic organ prolapse

Refer to pelvic organ prolaps quantitation (POP international quantitative framework) for grading method,^[13]^ which is divided into 5 degrees: Degree 0 without prolapse. Degree I, the lowest point of prolapse is above the hymen >1 cm (<−1 cm). Degree II, the lowest point of prolapse is above or below the hymen 1 cm (>−1 ∼ <1 cm); Degree III, the farthest part of prolapse is located in the subhymen >1 cm, but less than the vaginal length (>1 cm); Degree IV, the lower genital tract is completely everted, and the lowest point of prolapse is at least equal to the length of the vagina.

#### 2.6.2 Pelvic floor muscle strength measurement

PHENIXU4 neuromuscular stimulation therapy instrument was used to detect pelvic floor muscle strength of patients, including mean muscle strength (amplitude) and fatigue value (percentage reduction of muscle strength per second) of class I and II muscle fibers.^[14]^

#### 2.6.3 Evaluation of the curative effect of urinary incontinence

Urinary incontinence will be assessed using the International Incontinence Consultation Questionnaire.^[15]^

#### 2.6.4 Incidence of adverse reactions

Includes any treatment-related discomfort during treatment and follow-up.

### 2.7 Data collection and management

The data will be collected according to the evaluation criteria before the beginning of treatment, 4 weeks after the start of treatment and at the end of treatment. If patients drop out midway during the period, the reasons for withdrawal are recorded in detail, and the willingness of patients will be obtained if the data are used. Eight weeks after the end of treatment, each patient will be followed up by outpatient or telephone. The detailed records of the reasons for the loss of follow-up information cannot be collected. All data will be collected jointly by 1 or 2 assistants. Personal information of all patients will be collected, shared, and stored in a separate storeroom to protect the confidentiality before, during and after the test. Access to the database is limited to the researchers of this research group.

### 2.8 Statistical analysis

We will use SPSS.18 (SPSS Inc, Chicago, IL) software to analyze the research data. Fisher exact test and the Mann–Whitney *U* test will be performed for comparison of both groups, for categorical variables and continuous variables, respectively. When *P* <.05, the difference is statistically significant.

## 3 Discussion

There are mainly two kinds of pelvic floor muscles: type I and type II muscle fibers. Type I muscle fibers belong to slow contraction fibers, which play a supporting role in the pelvic cavity and are not prone to fatigue.^[16]^ Type II muscle fiber belongs to fast contractile fiber, which is an important component of pelvic and abdominal cavity's motor system. Its contraction is short and rapid, and is prone to fatigue.^[17]^ In the process of vaginal delivery, puerpera are easy to stretch and damage type I and II muscle fibers of the pelvic floor, resulting in urinary incontinence, pelvic organ prolapse, and so on.^[18,19]^ In early clinical practice, surgery is often used to achieve the purpose of treatment, but surgical resection of pelvic organs and tissues will not only cause greater damage to patients and slower recovery, but also easily lead to disease recurrence.^[20]^ Postpartum pelvic floor rehabilitation is an important link in the prevention and treatment of PFD. Studies have found that postpartum pelvic floor rehabilitation training can prolong the contraction duration of pelvic floor muscles, enhance the contractile force and contractile coordination ability of muscles, so as to effectively restore the pelvic floor muscle function.^[19]^ But the treatment cycle is long, and the requirements for patient compliance are high.^[21]^ Acupoint injection therapy for PFD has been used for a long time in China. By continuous stimulating drugs at specific acupoints, the efficacy of acupuncture can be strengthened and prolonged. Clinical studies have confirmed that Huang Qi has the effect of dilating blood vessels, improving microcirculation and nutritional status, and promoting the effect of nerve and muscle tissue repair.^[8]^ Modern studies have found that stimulation of Zusanli can increase the contractility of the uterus and surrounding tissues, and stimulation of Sanyinjiao can significantly increase the discharge times of electrical signals of the pelvic nerve, effectively improve the excitatory conduction of the pelvic nerve, and enhance the contractile force of the bladder detrusor.^[22]^ At present, there is no clinical study of acupoint injection combined with pelvic floor rehabilitation in the treatment of postpartum pelvic floor disorders. Therefore, we intend to evaluate its efficacy and safety through prospective randomized controlled trials. This study has the following limitations: due to the short follow-up time and the lack of evaluation of long-term efficacy maintenance, we may appropriately extend the follow-up time when necessary; at the same time, due to the influence of treatment, this study cannot achieve strict double-blind, which may affect the results.

## Author contributions

**Data collection:** Ying Zheng and Hongying Yang

**Data curation:** zheng ying, Hongying Yang.

**Funding acquisition:** Lihua Guo.

**Funding support:** Llihua Guo

**Investigation:** Hongying Yang.

**Resources:** Xunfu Yin and Xiujuan Ma

**Software operating:** Xunfu Yin and Xiujuan Ma

**Software:** Xunfu Yin, Xiujuan Ma.

**Supervision:** Hongying Yang.

**Writing – original draft:** Ying Zheng and Hongying Yang

**Writing – review & editing:** Ying Zheng and Llihua Guo
